# Simultaneous steering and imaging of magnetic particles using MRI toward delivery of therapeutics

**DOI:** 10.1038/srep33567

**Published:** 2016-09-26

**Authors:** Ouajdi Felfoul, Aaron T. Becker, Georgios Fagogenis, Pierre E. Dupont

**Affiliations:** 1 Boston Children’s Hospital/Harvard Medical School, Department of Cardiovascular Surgery, Boston, 02115, USA; 2 University of Houston, Department of Electrical and Computer Engineering, Houston, TX 77004-4005, USA

## Abstract

Magnetic resonance navigation (MRN) offers the potential for real-time steering of drug particles and cells to targets throughout the body. In this technique, the magnetic gradients of an MRI scanner perform image-based steering of magnetically-labelled therapeutics through the vasculature and into tumours. A major challenge of current techniques for MRN is that they alternate between pulse sequences for particle imaging and propulsion. Since no propulsion occurs while imaging the particles, this results in a significant reduction in imaging frequency and propulsive force. We report a new approach in which an imaging sequence is designed to simultaneously image and propel particles. This sequence provides a tradeoff between maximum propulsive force and imaging frequency. In our reported example, the sequence can image at 27 Hz while still generating 95% of the force produced by a purely propulsive pulse sequence. We implemented our pulse sequence on a standard clinical scanner using millimetre-scale particles and demonstrated high-speed (74 mm/s) navigation of a multi-branched vascular network phantom. Our study suggests that the magnetic gradient magnitudes previously demonstrated to be sufficient for pure propulsion of micron-scale therapeutics in magnetic resonance targeting (MRT) could also be sufficient for real-time steering of these particles.

While significant progress has been made in the development of cellular medicines and therapeutic drugs, techniques for their targeted delivery has not proceeded apace. While localized delivery is sometimes possible^1^, systemic injection remains the best option for deep-seated targets or for multiple targets dispersed through the body. Shortcomings of systemic administration, however, include the challenge of localizing the therapeutic at the desired location, limited circulation time due to filtering of the blood by the lungs, spleen, liver and kidneys and possible collateral damage when a therapeutic concentrates in an untargeted tissue.

Magnetic targeting during systemic delivery represents a promising approach to address these challenges as demonstrated by *in vitro*^2^,^3^,^4^ and animal studies^5^,^6^. In this approach, magnetic particles are incorporated into the therapeutic agent and the agent is concentrated at the targeted site via gradient fields generated by magnets. As an alternative to systemic circulation, a magnet can be used to steer a bolus of magnetized therapeutics through a bifurcation of the vasculature or bronchi^7^. The magnets are either placed on the skin^8^,^9^,^10^ or are surgically implanted^11^ near the targeted site. While permanent magnets or superconducting coils are typically used^8^,^9^,^10^,^12^, it is also possible to magnetize implanted ferromagnetic material, such as a stent, using a conducting coil placed around the body^13^.

While this approach enables localized targeting, it possesses some limitations. Gradient-induced forces decrease rapidly with distance from the magnet and it is also difficult to tailor the shape of the magnet’s gradient field to the anatomy. Furthermore, in systemic delivery, the magnet’s action is not to attract particles from other parts of the body, but rather to retain magnetized therapeutics passing in close proximity to it. In steering applications, delivery has been limited to guidance through a single bifurcation, such as to the left or right bronchi^7^. Furthermore, while a magnet placed on the skin of a mouse can generate a strong gradient in its deepest tissues, this is not true of the larger human body. Thus, targeting deep locations in humans requires the complications of surgical implantation and, if the implants are paramagnetic, the use of a magnetic coil system.

To address these limitations, magnetic resonance targeting (MRT) has been proposed as a favourable alternative to the use of skin-mounted or surgically implanted magnets. In this approach, the magnetic gradients of the scanner, normally used in imaging, are repurposed to produce magnetic targeting forces^3^,^14^,^15^,^16^,^17^. Since the gradients are generated uniformly throughout the bore of the scanner, forces can be produced everywhere inside the body and can be precisely controlled with respect to time. The magnitude of these forces is proportional to the magnetic particle volume as well as to the strength of the gradient field. To produce sufficiently strong gradients for effective nanoparticle targeting, prior demonstrations have employed either a small-bore research scanner^3^,^14^,^17^ or a clinical scanner with custom gradient coils^15^,^16^.

An important additional benefit of MRT is that the superparamagnetic nanoparticles used for MRI-based targeting also act as MRI contrast agents and so can be used to assess delivery^2^,^17^,^18^,^19^. Consequently, MRT enables the combined delivery and imaging of magnetically labelled therapeutics^3^,^14^,^15^,^16^,^20^. To fully exploit the dual roles of the MRI to both propel and assess delivery, however, pulse sequences are needed that enable the targeting forces to be adjusted in real time based on imaging of the magnetized therapeutic. With this capability, known as magnetic resonance navigation (MRN)^21^, it would be possible to steer a magnetized drug bolus through an arbitrary number of vascular bifurcations or, in the case of systemic injection, to ensure complete coverage of all targets in minimum scanner time.

The challenge of using MRI to both propel and image the magnetic particles, however, is that it has previously not been possible to do both at the same time. As a result, most demonstrations of MRT run an imaging sequence to assess delivery only after completion of the gradient-force steering sequence^3^,^14^,^15^,^16^. As with standard magnet-based targeting, this has limited steering demonstrations to navigation of a single vascular bifurcation^3^,^15^,^16^.

One approach to achieving MRN is to rapidly alternate between particle imaging and propulsion. To achieve fast particle localization, a projection technique is used that images only the particles and not the surrounding tissue^22^. This technique has been demonstrated in vitro using superparamagnetic nanoparticles^14^ and *in vivo* for real-time millimetre-scale particle navigation inside a porcine carotid artery^21^. It has also been employed to control MRI-powered needle driving robots^23^. Since MR imaging is relatively slow, however, this approach requires high propulsive forces to compensate for the non-propulsive time spent imaging. To achieve such forces high-gradient scanners were used^14^ or millimetre-scale particles were used and blood flow was blocked using a balloon catheter^21^.

In this report, we describe an alternate approach to MRN in which a single MRI pulse sequence simultaneously images and propels ferromagnetic material. The sequence allows a tradeoff between imaging frequency and maximum propulsive force. For example, at an imaging rate of 52 Hz, the maximum force equals 90% of the maximum force that can be applied when no particle imaging is performed. In contrast, the prior approach of alternating between imaging and propulsion would result in no net force at this imaging rate. The proposed sequence generates a projection image of particle location along the desired propulsion direction and is shown to provide accurate position measurements at imaging frequencies over 100 Hz. The propulsion and imaging capabilities of the sequence are experimentally validated on a clinical scanner and the capability for real-time steering is demonstrated in the context of a millimetre-scale particle performing high-speed navigation of a multi-branched tube network.

## Results

**Figure 1:**
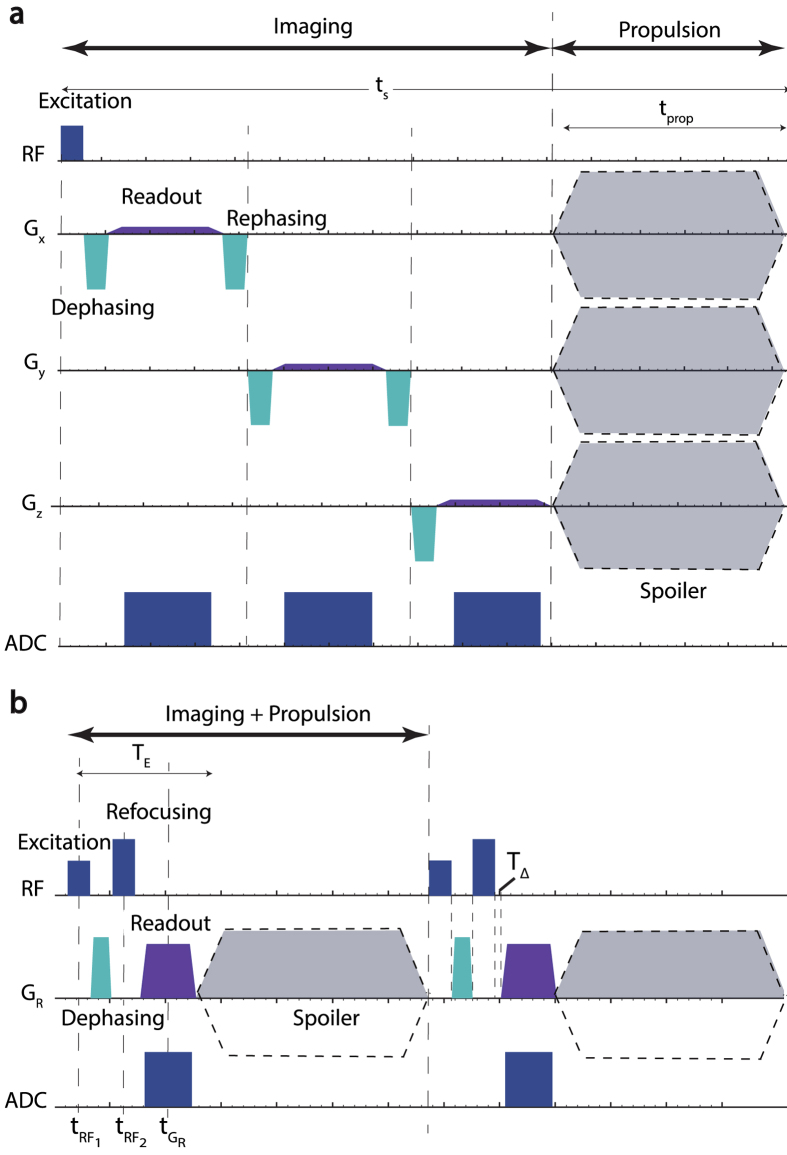
MRI pulse sequences for imaging and steering. (**a**) Alternating imaging and propulsion. Imaging is performed along the three axes of the MRI coordinate frame using a gradient echo pulse sequence. (**b**) Proposed simultaneous imaging and propulsion. Imaging and propulsion are performed along a single desired direction. *RF* = Radio Frequency excitation, *ADC* = Analogue to Digital Converter Sampling.

**Figure 2:**
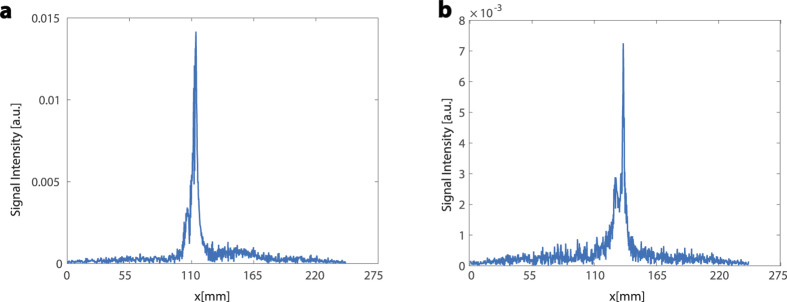
Examples of MRI-based projections used for particle localization: (**a**) 50 Hz, (**b**) 117 Hz.

To design a pulse sequence that simultaneously images and propels, we first studied the structure of existing pulse sequences that alternately image and propel (Fig. 1a)^21^,^23^. In these sequences, positive contrast projections (see Fig. 2) of ferromagnetic material^22^,^24^ are obtained through a combination of radio frequency (RF) pulses and magnetic gradient pulses (*G*_*x*_, *G*_*y*_, *G*_*z*_) applied in the three directions of the MRI coordinate frame. These magnetic gradients also produce propulsive forces on ferromagnetic material in the corresponding coordinate directions. Since the magnitudes required for imaging are typically small and the pulses are of both positive and negative sign, the imaging gradients produce little propulsive force and in some cases have been designed to exactly cancel^14^. Following imaging, propulsion is accomplished using gradient pulses of varying amplitude and duration. In the context of standard MRI pulse sequences, the propulsive gradients take the place of spoiler gradients which are traditionally intended to clear the effect of the preceding imaging pulses in preparation for acquiring the next image.

To optimize image-based steering, we investigated the design of pulse sequences that would maximize two criteria. The first is the propulsive duty cycle defined by the fraction of the total pulse duration during which propulsion can occur. The second criteria is the maximum possible propulsive force (averaged over the pulse sequence) normalized by the force corresponding to the maximum gradient applied for the entire duration of the pulse sequence.

To maximize duty cycle, we first observe that some important steering tasks do not require real-time particle position in all three dimensions, but rather only in the direction of propulsion. For example, when steering through the vasculature, particles move with the blood flow and the goal of propulsion is to navigate the bifurcations. This involves steering particles perpendicular to the flow toward the desired branch. Standard MRI imaging can provide a 3D map of the vasculature. Since the image of the vasculature is created with respect to the MRI frame, no additional image registration step is required. A bolus of injected particles can be tracked in this map by imaging along the axis of the vessel. As a bifurcation is approached, the pulse sequence can switch to directing the particles into the desired branch. Imaging in the steering direction enables monitoring the progress of the bolus as it passes through the bifurcation. Once past the bifurcation, the process of particle tracking and steering can be repeated until the target site is reached.

Based on this observation, we designed the pulse sequence such that the imaging direction coincides with the direction of the gradient pulses and, furthermore, such that all the gradient pulses are of the same sign so that they do not cancel each other out. In this way, imaging gradients contribute in their entirety to propulsion. Spin echo imaging sequences can be adapted to provide these properties as shown in Fig. 1b. Note that the propulsion/imaging direction can be selected arbitrarily in {*x*, *y*, *z*} space, can vary from instant to instant, and is not constrained to align with one of the MRI coordinate axes.

To enable maximum propulsive force, we tuned the overall pulse sequence to maximize the amplitude of the dephasing and readout gradient pulses and to minimize total sequence duration (see Pulse Sequence Design in Methods). As implemented on a standard Siemens 3T Skyra scanner, we achieved good image quality for the magnetic particles using dephasing and readout gradient magnitudes of 20.85 and 19.6 mT/m (scanner maximum is 21 mT/m) with a pulse duration of 5.5 ms.

We include a spoiler as shown in Fig. 1b to control the imaging frequency as well as the amplitude of the average propulsion force. Imaging frequency is controlled by adjusting the duration of the spoiler. The maximum possible imaging frequency corresponds to a spoiler of duration zero. For the parameters used in the examples, the maximum is 1/0.0055 = 182 Hz.

Spoiler amplitude and sign are used to control average propulsive force. For some steering applications, we may wish to apply a bang-bang control strategy consisting of applying the maximum propulsive force at every time instant. In these cases, the spoiler can be selected to be of maximum amplitude and of the same sign as the imaging gradients. In other situations, we may wish to apply less than the maximum propulsion force or even no force. This can be accomplished by applying the spoiler amplitude that produces the desired average force over the duration of the pulse sequence. Note that providing the capability to image without propulsion (produce zero net force) requires that the spoiler cancel the propulsive effect of the imaging gradients. This imposes a minimum spoiler duration and reduces the maximum imaging frequency. For the parameters considered here, the minimum spoiler duration is 3.4 ms and the maximum imaging frequency is reduced to 112 Hz.

### Maximum Average Force versus Imaging Frequency

To validate our proposed pulse sequence, we implemented it on a Siemens 3T Skyra scanner and measured the maximum average force as a function of imaging frequency. In these experiments, spoiler gradient amplitude was set to the scanner maximum of 21 mT/m and the sequence was applied so as to generate horizontal forces on a pendulum comprised of a 6 mm chrome steel sphere suspended from a 200 mm string. Pendulum angle was used to compute average force.

**Figure 3:**
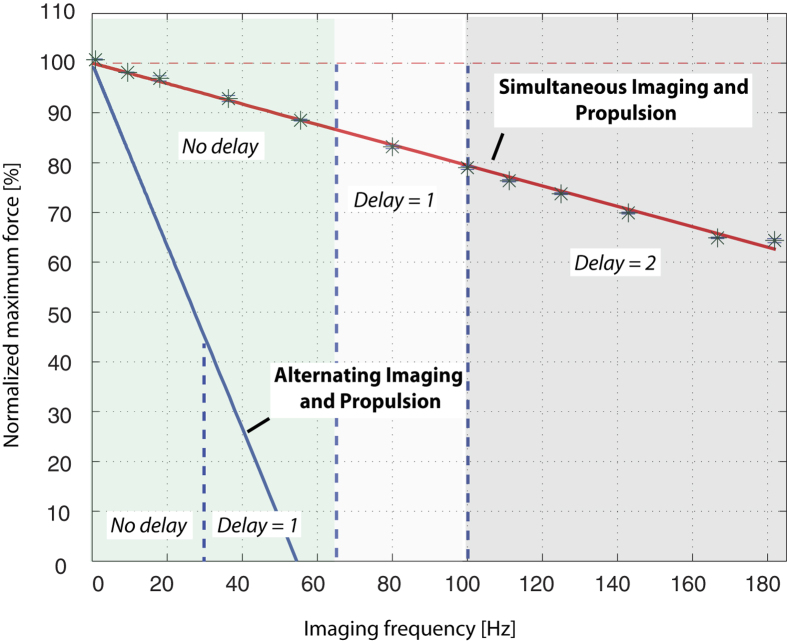
Maximum average force produced by sequences of Fig. 1 normalized by the maximum possible force that can be produced by the scanner. Experimental data points are denoted by *. Dashed vertical lines separate frequency ranges according to the delay, measured in number of pulse sequences, between when an image is taken and when it is available for modulating propulsive force.

Predicted and measured maximum average force for the sequence of Fig. 1b are plotted in Fig. 3 normalized by the force associated with a constant maximum gradient of 21 mT/m. The equivalent result for the interleaved imaging and propulsion Fig. 1a is also shown. With alternating imaging and propulsion, the maximum normalized force falls to less than 50% at a control frequency of 27 Hz and reaches zero at about 52 Hz. In contrast, the proposed spin echo sequence provides 95% of the open-loop force at 27 Hz and 63% of open-loop force at 182 Hz.

In steering applications, the image acquired during each pulse sequence is used to determine the force direction and magnitude of subsequent pulse sequences. Since a finite amount of time is needed to process the image and load the next sequence into the scanner (10 ms in our implementation), there can be a delay between when an image is taken and when it can be applied. As indicated by the dashed lines in Fig. 3, the delay depends on imaging frequency. With our simultaneous imaging and actuation sequence, for frequencies under 64 Hz, there is no delay and image data from one pulse sequence can be immediately applied in the next pulse sequence. In contrast, alternating imaging and propulsion introduces no delay only for frequencies under 35 Hz and a delay of one pulse sequence for higher frequencies. Below 100 Hz, simultaneous imaging and propulsion introduces a delay of one pulse sequence since the data can be applied two pulse sequences later. For higher frequencies, there is a two pulse sequence delay.

### Image-based Position Error versus Imaging Frequency

To investigate the effect of imaging frequency on particle position error, we used our pulse sequence to apply a sinusoidal force to a 2.34 mm diameter chrome steel sphere submerged in semi-cylindrical water-filled channel. The propulsive force was directed along the channel causing the bead to roll back and forth along the bottom of the channel. A high-speed video camera placed outside the scanner recorded particle motion at 240 fps and was used as a baseline for evaluating MRI-based position measurements.

**Figure 4:**
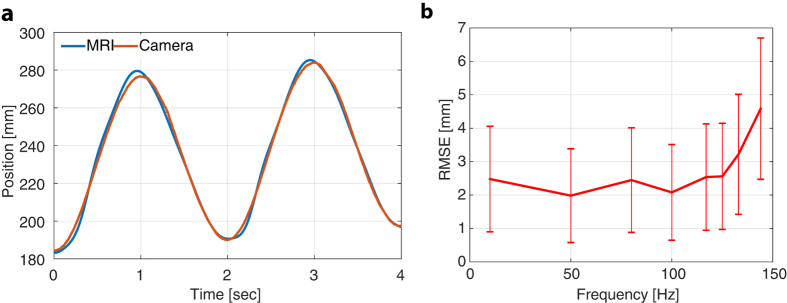
Image-based position measurement. (**a**) Particle position versus time as estimated by MRI and high-speed camera for an MRI imaging frequency of 125 Hz. (**b**) Error in MRI-based position measurement as a function of imaging frequency. RMSE = Root Mean Square Error.

With a sinusoidal forcing frequency of 0.5 Hz and a displacement amplitude of 100 mm, the maximum particle velocity in these experiments was about 314 mm/s, which is the same order of magnitude as blood velocity in the ascending aorta (peak and mean velocities of 66 and 11 cm/s^25^). An example plot for an imaging frequency of 125 Hz is given in Fig. 4(a). As shown in the plot of position error versus imaging frequency in Fig. 4(b), mean position error is slightly larger than particle diameter for frequencies up to 125 Hz. Errors of this size are acceptable for image-based steering as demonstrated in the following section.

### Image-based Particle Steering

**Figure 5:**
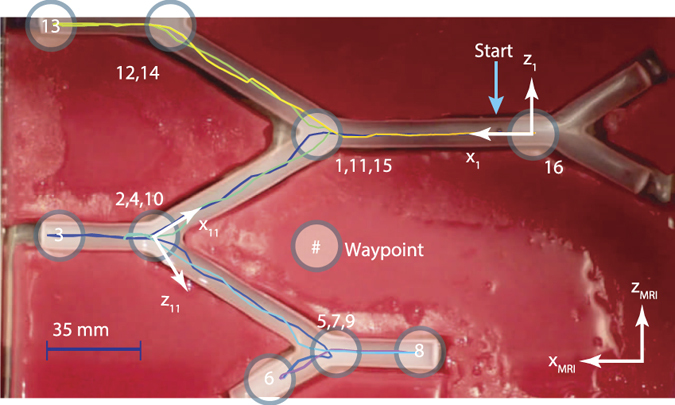
Fluid-filled vascular network phantom for image-based particle steering. Sequence of 16 waypoints for 2 mm diameter sphere are labeled. Image coordinate frame indices correspond to the target waypoint, *i.e.*, frame *k* is defined at waypoint (*k* − 1) with its *x*-axis pointing in the scan direction toward waypoint *k*. Particle path between waypoints for an MRI imaging frequency of 117 Hz is shown and video of particle motion is provided in the Supplementary Movie.

To illustrate the use of simultaneous imaging and propulsion for active steering, experiments were conducted using the fluid-filled vascular network phantom of Fig. 5. A sequence of 16 waypoints was defined as shown and the MRI scanner was programmed to apply pulse sequences that would propel a 2 mm diameter chrome steel sphere through the sequence of waypoints as fast as possible (see methods). It was determined experimentally that 117 Hz was the highest imaging frequency that could reliably generate sufficient force to propel the particle through a combination of rolling and sliding along the bottom of the fluid-filled channel. The Supplementary Movie depicts the particle motion.

**Figure 6:**
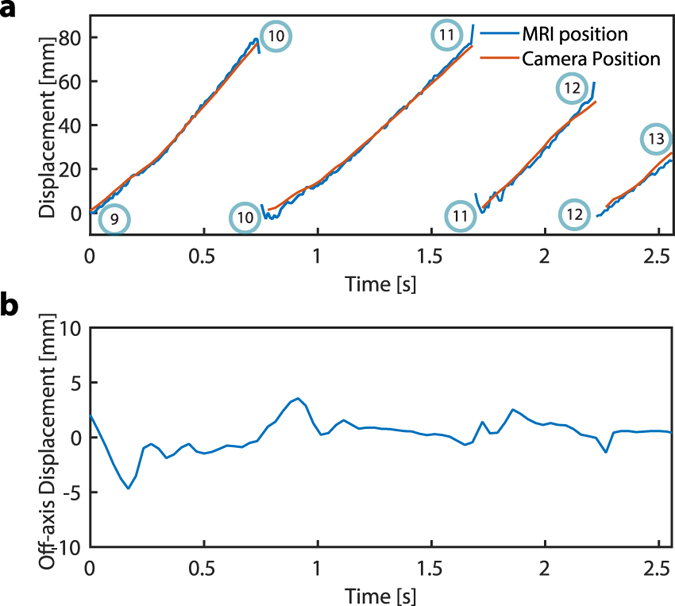
Particle displacement versus time for motion between waypoints 9, 10, 11, 12 and 13. (**a**) Displacement along channel. (**b**) Displacement perpendicular to channel.

Figure 6 illustrates video camera and MRI displacement data for the particle trajectory connecting waypoints 9, 10, 11, 12, and 13. Since the proposed MRI sequence only measures position in the direction of propulsion, the two dimensional motion of the particle is plotted along axes parallel and perpendicular to the channel segments connecting adjacent waypoints. Particle motion parallel to the channel reaches an average velocity of about 74 mm/s (peak velocity is about 110 mm/s). For these segments, MRI position error relative to the camera was 7 mm ± 14.76 mm. Motion perpendicular to the channel is measured only by the video camera and is shown in Fig. 6(b). This plot shows that the particle experiences small-amplitude damped oscillations about the centre of each channel. Note that since the axis of motion is redefined as the particle reaches each waypoint, there are discontinuities in the plot at these times.

## Discussion

In this report, we have proposed a technique for MRN in which gradient pulses simultaneously image and propel ferromagnetic particles. This approach avoids the limitation of prior methods that stopped propulsion during imaging. The result is a substantial increase in both the maximum force and imaging frequency that can be achieved during image-based steering. Using this technique, we have demonstrated high-speed real-time steering of a millimetre-scale ferrous particle through a vascular network phantom. This was performed in a standard clinical scanner using pulse sequence code written in the native scanner software. While this report has not considered specific *in vivo* flow conditions, prior work^21^ has demonstrated that MRI scanners are capable of propelling ferrous particles in the bloodstream of live animals. The proposed technique should provide improved *in vivo* results since it can produce higher forces and imaging rates than prior pulse sequences.

Applying this method to clinical applications involving real-time navigation of magnetically-labelled cells and drug particles depends on (i) producing sufficient force on these smaller particles, and (ii) being able to image small collections of particles. Gradients of 300 mT/m are sufficient for propulsion at this scale^15^ and gradient coils producing such magnitudes have already been demonstrated in both customized^16^ and uncustomized^26^ clinical scanners. Furthermore, the post-propulsion verification imaging of distributions of particles and cells employed in MRN^3^,^14^,^15^,^16^,^17^ provides promise that real-time image-guided navigation will become possible.

## Methods

### Particle Imaging and localization

Imaging was performed using the ferromagnetic particle’s induced magnetic field as a slice-select gradient to acquire a positive contrast projection^22^,^24^ in the desired propulsion direction. In standard MR imaging, an image coordinate system is defined in terms of three orthogonal directions and the gradients applied in these directions. These are termed the slice-select (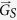), phase-encode (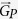) and readout gradients (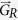), which satisfy 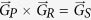. They are related to the physical coordinates of the scanner (*i.e.*, MRI-coordinate frame) by a rotation matrix. 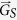 is used to define the desired imaging plane orthogonal to the slice-select vector. 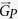 selects a line in the image plane orthogonal to the phase-encode direction while the 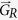 creates a frequency domain encoding of the points along that line. By sequentially sampling all lines in the plane, an image is reconstructed via an inverse 2D Fourier transform.

This method can be simplified to acquire a positive contrast projection of a ferromagnetic particle^22^,^24^. Rather than apply a slice-select gradient to select an imaging plane, the magnetic field produced by the ferromagnetic material is used to select a volume surrounding the particle and so 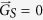. By the projection-slice theorem, the projected image of the excited volume onto a line parallel to readout axis can be obtained as the inverse Fourier transform of the image-space line passing through the origin. This image-space line through the origin is selected by setting the phase-encode gradient to zero, 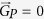. Thus, obtaining a projection along the readout axis requires only applying a gradient in that direction as shown in Fig. 1.

To obtain particle position, a projection is first acquired when the particle is at a known location along any axis; this projection is used as a correlation mask. For each subsequent projection, we compute the convolution of the correlation mask with the current projection and detect the peak in the resulting signal. The peak corresponds to the displacement of the particle relative to the initial position. By shifting the correlation mask to the isocenter of the scanner, the displacement of the particle is given relative to the centre of the scanner bore.

Projection data was collected using a clinical coil for spinal imaging comprised of thirty-two antennas. In the image-based particle steering experiments, an CNR-weighted average of antenna signals was used in computing particle position. In the experiments measuring position error as a function of imaging frequency, the particle oscillation amplitude was 10 cm resulting in only two antennas being excited, with the quality of signal in each depending on particle position. For channel selection, we developed a localization algorithm similar to a Kalman filter that works as follows: given the current position of the bead and a velocity estimate computed using prior position estimates, the algorithm computes a probability distribution describing particle position for the next time instant with its mean based on current velocity model and its variance defined by its maximum possible velocity. Next, particle position is computed from the projection data for each antenna as described above. Particle position is then computed using the predicted position distribution to determine which antenna measurement is more likely to be correct. If both antenna measurements lie below a threshold, particle position is set to the predicted constant-velocity value. Finally, the position estimate is filtered recursively using exponential smoothing.

Video cameras mounted on a tripod just outside the 5-Gauss line were used to record particle motion via an angled mirror mounted inside the scanner bore. For the experiments reporting image-based position error versus imaging frequency, the image pixel size was 0.27 by 0.27 mm (iPhone 6, 12X telephoto lens, 240 fps). For the image-based particle experiments, the image pixel size was 0.19 by 0.19 mm (Canon T1i, 30 fps). Video frames went through basic image processing to extract the particle’s position. Initially, each colour frame was converted from RGB to grayscale. Next, a histogram equalization step was performed to increase contrast. Finally, the image was converted to binary by thresholding at a 30%-level of gray-value range. The binary image was the input to a Hough circle detection algorithm. The centre of the detected circle was the position of the bead. At particular frames when circle detection fails (e.g., due to glare), the position of the particle is updated based on a constant-velocity model. The above is equivalent to a Kalman filter with a constant-velocity model as the process update and circle detection as the measurement update step.

### Pulse Sequence Design

The simultaneous imaging and propulsion pulse sequence of Fig. 1b was implemented on a Siemens Skyra 3T scanner using the IDEA user development kit. Parameters are given in Table 1. The pulse sequence is comprised of RF excitation and refocusing pulses as well as dephasing and readout gradients. While these components are required for imaging, the spoiler gradient is optionally used in imaging to cancel unwanted signals from the previous excitation. For MRN, the spoiler gradient can be used to control propulsive power.

To maximize the propulsion obtained from the imaging gradients, the gradient magnitudes should be maximized and their total duration should be minimized while still achieving acceptable image quality. In this case, the sequence was designed to provide a spatial resolution of 0.56 mm.

Since the RF and gradient components required for imaging cannot overlap in time, the minimum length pulse sequence is obtained by eliminating any time gaps between pulses as shown. The one constraint is that the overall pulse sequence must generate an echo signal at the center of the readout gradient requiring that


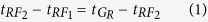


with 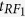, 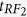 and 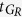 corresponding to the mid-point time of the excitation pulse, the refocusing pulse and the readout gradient. This requires the insertion of the depicted delay between the refocusing pulse and the readout pulse. Specific parameter values can be selected as described below.

First, the readout field of view, *L* = 250 mm, and the base resolution, *n* = 448, are selected according to the phantom size and the desired spatial resolution. The readout gradient amplitude and its duration, 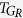 are then given by


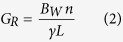



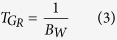


where the gyromagnetic ratio 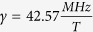, and *B*_*W*_ is the receiver bandwidth per pixel. The value *B*_*W*_ describes the mapping of frequency range from the analysed echo signal to each pixel.

Since increasing *B*_*W*_ increases readout gradient amplitude and decreases its duration, the optimal solution is to select *B*_*W*_ such that *G*_*R*_ = *G*_max_ where *G*_max_ is the largest magnitude of gradient that can be produced by the scanner. Increasing *B*_*W*_, however, also decreases the signal-to-noise ratio of the projection images. Thus, the largest acceptable value of *B*_*W*_ must be determined experimentally. For the experiments described here, acceptable imaging quality was achieved using *B*_*W*_ = 470 Hz/pixel which resulted in *G*_*R*_ = 19.6 mT/m and 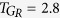 ms. For the Siemens Skyra 3T scanner used, the readout gradient was slightly less than the maximum gradient of *G*_max_ = 21 mT/m.

The magnitude and duration of the dephasing gradient can be selected based on the readout gradient to satisfy


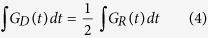


To avoid safety limits associated with operating the gradient coils at maximum current, *G*_*D*_ was selected as *G*_*D*_ = 20.85 resulting in 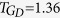 ms.

The duration of the excitation and refocusing RF pulses, 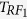 and 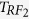 are normally selected to optimize image spatial resolution. For the examples considered here, durations of 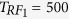 *μ*s and 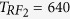 *μ*s were experimentally determined to be the shortest pulses that provide adequate image quality.

Finally, to satisfy equation (1), a delay must be inserted between the refocusing pulse and the readout pulse. Since the duration of the dephasing gradient is approximately half the duration of the readout gradient, the duration of the delay, *T*_Δ_ = 0.23 ms, is equal to approximately half of the duration of the RF excitation pulse, 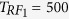 *μ*s.

### Force as a Function of Imaging Frequency

To experimentally measure the maximum force generated by the pulse sequence, a pendulum, comprised of a 6 mm radius chrome steel sphere and 200 mm long thread, was suspended from the top of the MRI bore. The thread was considered massless since it is less than 0.3% of the mass *m* of the bead. At equilibrium, the angle *θ*_*p*_ of the pendulum is given by:


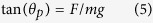


where *F* is the time-averaged magnetic force and *g* is the gravitational acceleration (9.8 *m*/*s*^2^). The pendulum was recorded with a video camera, and the angle *θ* was computed from sphere position by averaging over 5 seconds of video data recorded at 30 fps. The maximum oscillations around equilibrium angles were measured to ±1° with and without the magnetic force applied.

### Vascular Phantoms

All phantoms were comprised of 12 mm-diameter semicylindrical tubes 3D-printed from VeroWhite on a Stratsys Object30. The tubes were filled with water and embedded in a 1% w/v agar preparation (Agarose HS, Denville Scientific Inc.). The agar was used to generate sufficient MR signal for imaging and to hold the tubing network in place. A red colourant was added to the agar to increase the contrast between the tube and the background.

### Image-based Navigation (MRN)

The MRI gradient coils produce a magnetic field **B**_*g*_(*t*) that exerts on a ferrous particle the force


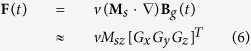


Here *v* is the volume of the magnetic material, **M**_*s*_ is its saturation magnetization and *M*_*sz*_ is the *z* component of magnetic saturation. In the above equation, **M**_*s*_ ≈ *M*_*sz*_ since the strong magnetic field along an MRI bore dominates off-axis magnetization. Because the MRI has a set of three separate orthogonal gradient coils producing gradients *G*_*x*_, *G*_*y*_ and *G*_*z*_, arbitrarily directed forces can be applied to any ferromagnetic body inside the scanner.

Closed-loop control can be implemented using a discrete time version of equation (6),


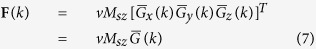


Here, *k* represents the pulse sequence index and 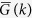 is the vector of mean coordinate-direction gradients over the sequence,


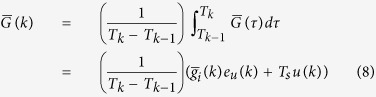


where *T*_*k*_ is the time at the end of pulse sequence *k*, 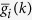 is the portion of the integral associated with imaging gradients and *T*_*s*_ is the duration of the spoiler gradient. The spoiler gradient vector is 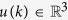 and the unit vector *e*_*u*_(*k*) = *u*(*k*)/||*u*(*k*)|| ensures that the imaging gradients are aligned with the spoiler gradient. Here, *u*(*k*) also acts as the control input vector which is selected based on the navigational task.

For the experiments involving sinusoidal motion in a straight channel, the control input vector *u*(*k*) was selected as


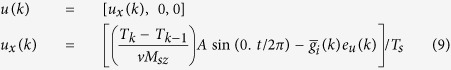


in which *A* was selected experimentally to provide a peak-to-peak amplitude of 100 mm.

For the image-based navigation experiments in the vascular network phantom, the relative direction of each branch of the network was known and a registration procedure was used to measure the orientation of the network in the plane of the scanner bed. In an actual intervention, network mapping could be performed using preoperative images and registration could employ standard algorithms.

To perform MRN, the propulsive force direction was selected as the direction of the next desired waypoint, 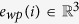, ||*e*_*wp*_(*i*)|| = 1, in which *i* is the index of the next waypoint. To maximize particle speed, the force magnitude was set to its maximum. Thus, the control input vector was selected as follows.


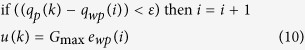


in which current particle position is given by 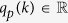 and 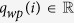 is waypoint position, both measured along *e*_*wp*_(*i*). The parameter *ε* > 0 determines the radius of the switching zone surrounding a waypoint and *G*_max_ is the maximum gradient amplitude of the scanner.

## Additional Information

**How to cite this article**: Felfoul, O. *et al*. Simultaneous steering and imaging of magnetic particles using MRI toward delivery of therapeutics. *Sci. Rep.* **6**, 33567; doi: 10.1038/srep33567 (2016).

## Supplementary Material

Supplementary Information

Supplementary Movie 1

## Figures and Tables

**Table 1:** Pulse sequence parameters.

Parameter (units)	Symbol	Value
Readout Field of View (mm)	L	250
Base resolution (points)	n	448
Receiver Bandwidth per pixel (Hz/pixel)	*B*_*W*_	470
Maximum gradient amplitude (mT/m)	*G*_max_	21.00
Excitation RF flip angle (°)	*α*	90
Excitation RF duration (μs)	*T*_*RF*1_	500
Refocusing RF duration (μs)	*T*_*RF*2_	640
Readout gradient duration (ms)	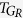	2.76
Readout gradient amplitude (mT/m)	*G*_*R*_	19.57
Dephasing gradient duration (ms)	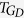	1.36
Dephasing gradient amplitude (mT/m)	*G*_*D*_	20.85
Time delay (ms)	*T*_Δ_	0.23
Minimum Imaging Sequence duration (ms)	*T*_*Seq*_	5.5
