# Supplementary material for: Simultaneous steering and imaging of magnetic particles using MRI toward delivery of therapeutics
Source: Sci Rep. 2016 Sep 26;6:33567. doi: 10.1038/srep33567 (PMC5036040; doi:10.1038/srep33567)
Supplement: Supplementary Information [file srep33567-s1.pdf]

Supplementary Materials for

Simultaneous steering and imaging of magnetic particles using MRI toward delivery of therapeutics

Ouajdi Felfoul, Aaron T. Becker, Georgios Fagogenis and Pierre E. Dupont

correspondence to: pierre.dupont@childrens.harvard.edu

This PDF file includes:

Caption for Movie: “Simultaneous steering and imaging of magnetic particles using MRI”

Other Supplementary Materials for this manuscript includes the following:

Movie: “Simultaneous steering and imaging of magnetic particles using MRI”

Movie: "Simultaneous steering and imaging of magnetic particles using MRI"

Caption for movie: This video depicts a 2mm diameter steel sphere moving between a set of 16 waypoints inside a fluid-filled vascular network phantom. The proposed pulse sequence is being used to image sphere position at 117Hz while simultaneously propelling it toward the next waypoint. When the particle enters a neighborhood of the current waypoint, the scanner detects this and begins propelling the particle toward the next waypoint.
